# A machine learning framework for predicting shear strength properties of rock materials

**DOI:** 10.1038/s41598-025-91436-8

**Published:** 2025-03-13

**Authors:** Daxing Lei, Yaoping Zhang, Zhigang Lu, Guangli Wang, Zejin Lai, Min Lin, Yifan Chen

**Affiliations:** 1 School of Resources and Civil Engineering, Gannan University of Science and Technology, Ganzhou, 341000 China; 2 Key Laboratory of Intelligent and Green Development of Tungsten & Rare Earth Resources, Provincial Department of Education, Ganzhou, 341000 Jiangxi China; 3 Anhui Lujiang Longqiao Mining Co., LTD, Hefei, 230000 China; 4 School of Resources and Safety Engineering, Central South University, Changsha, 410083 Hunan China

**Keywords:** Rock materials, Machine learning, Shear strength, Internal friction angle, Cohesion, Extreme gradient boosting (XGBoost), Civil engineering, Solid Earth sciences

## Abstract

The shear strength characteristics of rock materials, specifically internal friction angle and cohesion, are critical parameters for the design of rock structures. Accurate strength prediction can significantly reduce design time and costs while minimizing material waste associated with extensive physical testing. This paper utilizes experimental data from rock samples in the Himalayas to develop a novel machine learning model that combines the improved sparrow search algorithm (ISSA) with Extreme Gradient Boosting (XGBoost), referred to as the ISSA-XGBoost model, for predicting the shear strength characteristics of rock materials. To train and validate the proposed model, a dataset comprising 199 rock measurements and six input variables was employed. The ISSA-XGBoost model was benchmarked against other models, and feature importance analysis was conducted. The results demonstrate that the ISSA-XGBoost model outperforms the alternatives in both training and test datasets, showcasing superior predictive accuracy (R² = 0.982 for cohesion and R² = 0.932 for internal friction angle). Feature importance analysis revealed that uniaxial compressive strength has the greatest influence on cohesion, followed by P-wave velocity, while density exerts the most significant impact on internal friction angle, also followed by P-wave velocity.

## Introduction

**Fig. 1:**
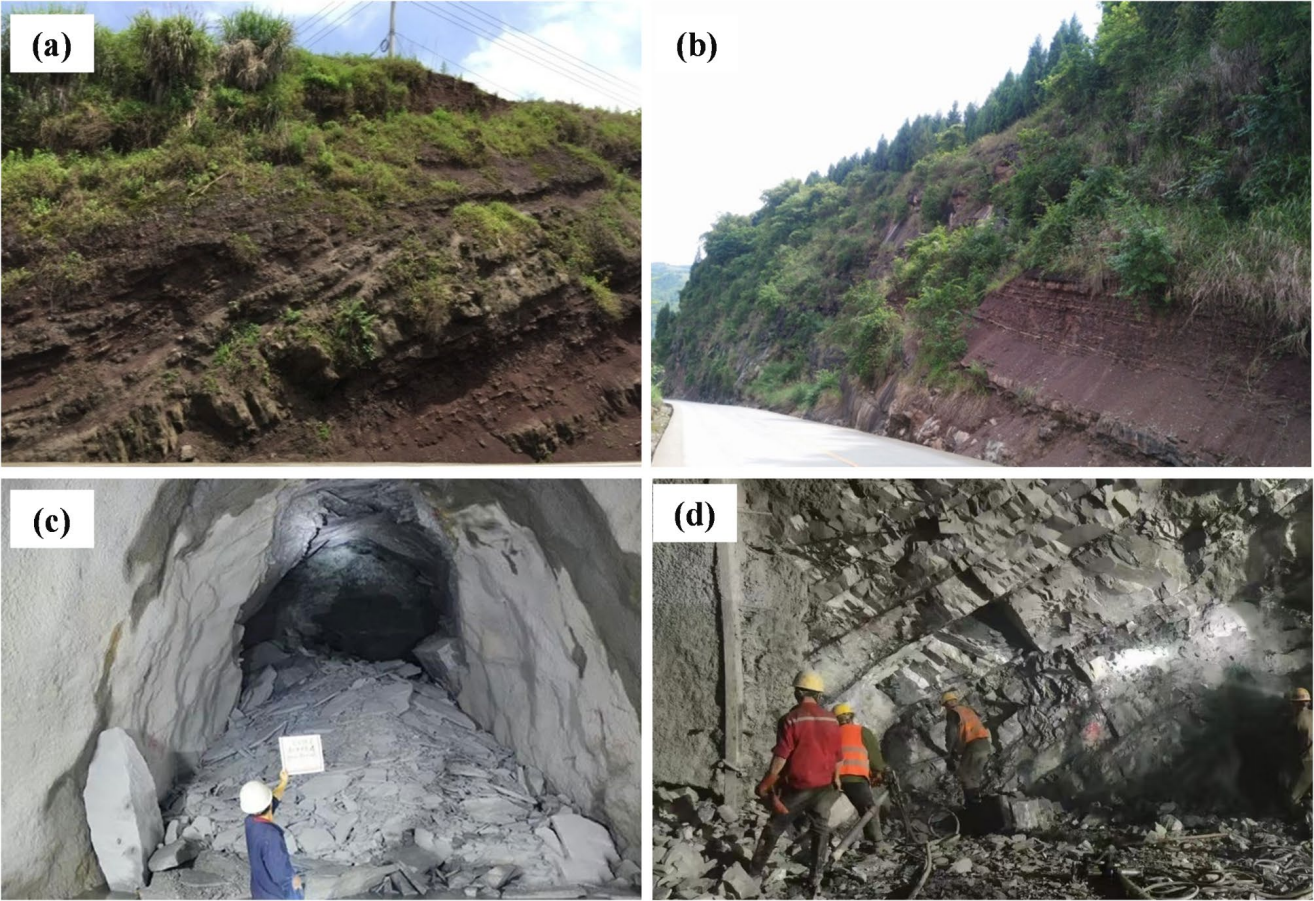
Photograph of (**a**) slope of mining surface, (**b**) slope of mountainous highway, (**c**) underground cavern rock engineering, (**d**) underground mining engineering in China, (photograph by Daxing Lei).

The mechanical properties of rock materials are a fundamental focus for geotechnical engineers and geologists involved in rock engineering^1–3^. Among these properties, the internal friction angle (*φ*) and cohesion (*c*) are the primary parameters defining the shear strength of rock materials^4,5^. They are critical for the rational design and safe operation of engineering applications, including rock slopes, underground chambers, and foundations (see Fig. 1). For example, using the wrong internal friction angle and cohesion can lead to overestimation or underestimation of the failure probability of rock materials^6^. Therefore, reasonable and accurate prediction of shear strength characteristics is helpful to reduce the construction risk and provide sufficient countermeasures for the design of engineering^7–9^.

Traditionally, cohesion and internal friction angle are obtained by testing standard specimens using triaxial compression equipment^10^. This method is widely regarded as the most accurate and is universally accepted. Over the years, numerous experts have significantly advanced the understanding of cohesion and internal friction angle in rock materials^11–13^. Through extensive experiments, numerical analyses, and theoretical approaches, researchers have explored the mechanical behavior of rock materials, resulting in a wealth of published studies^14–21^. For instance, Gu et al.^22^ investigated the evolution of mechanical parameters such as deformation modulus, internal friction angle and cohesion of Shuangjiangkou granite under different stress paths. Similarly, Hashiba and Fukui^23^ examined the loading-rate dependence of force and internal friction angle from a small amount of rock sample. However, experimentalists often face safety risks from close observation, and test results are susceptible to biases caused by unfavorable field conditions. In practice, due to the associated cost and time requirements (such as the early stages of a project), geotechnical engineers need to evaluate *φ* and *c* without triaxial test results^24,25^. To address these limitations, researchers have sought to develop inexpensive and efficient indirect estimation methods. Many researchers^26–33^ have explored the use of parameters such as uniaxial compressive strength (UCS), uniaxial tensile strength (UTS), sound velocity, gamma-ray data, and porosity to estimate *φ* and *c* in the absence of triaxial test results. Nevertheless, the complex and highly nonlinear relationships between these influencing factors and strength characteristics pose significant challenges. Traditional regression models, constrained by their reliance on fitting methods, can only account for a limited number of variables and fail to capture the intricate interdependencies among multiple factors.

Machine learning (ML) technologies offer high efficiency and precision, making them well-suited for analyzing the nonlinear and complex relationships among multiple input parameters^34–39^. ML-based predictive models not only substantially reduce experimental workload but also outperform regression methods in handling regression problems with significantly higher accuracy^40–44^. The application of ML in modeling complex problems has been extensively validated^45–49^. For example, based on the genetic programming (GP), Shen and Jimenez^24^ applied GP to predict the internal friction angle and cohesion of sandstone in the absence of triaxial data. The results show that the proposed ML model can provide good prediction performance. Taking the P-wave velocity of rock samples as the input parameter, Kainthola et al.^15^ applied linear regression analysis and adaptive neurofuzzy inference system (ANFIS) technology to establish an ML model of rock materials. Similarly, Hiba et al.^50^ employed a neural network model to predict *φ* and *c* from the logging data of two existing wells. Their study also included sensitivity analyses for three input parameters—neutron porosity (NPHI), compressional time (DTC), and bulk density (ROHB)—to assess their relative importance. Table 1 summarizes studies on the initial applications of ML methods for predicting cohesion and internal friction angle. Nonetheless, ML-based methods have not yet been widely used to predict cohesion and internal friction angle^51^. In limited research, scholars have adopted ML technologies such as artificial neural network (ANN), particle swarm optimization (PSO) and ANFIS to establish ML models and preliminarily prove their feasibility. However, existing research has mainly used some straightforward and ML algorithms, while the applicability of more advanced algorithms, such as the integrated algorithms of XGBoost and improved sparrow search algorithm (ISSA), in evaluating internal friction angle and cohesion has not been explored.

**Table 1:** ML models for the estimation of cohesion and internal friction angle.

Model	Parameters	Rock materials	Performance
ANN^52^	Drilling rate of penetration ROP; Weight on bit WOB; Drill pipe pressure SPP; Torque; Drilling fluid pumping rate	/	Correlation coefficient (R) values were 0.85 and 0.89 for friction angle and cohesion respectively
ANN^50^	Bulk density (ROHB), compressional time (DTC), and neutron porosity (NPHI)	Carbonate	Average absolute percentage error (AAPE) = 1.1% for the friction angle and AAPE = 2.4% for the cohesion.
Genetic algorithm (GA)-ANN^53^	P-wave velocity, uniaxial compressive strength and brazilian tensile strength	Limestone	R^2^ 0.967 (*c*)
GP^24^	UCS, UTS, σ_3_	Sandstone	/
ANFIS^54^	P-wave velocity	Limestone, quartzite, slate and quartz mica schist.	/
ANFIS-PSO^55^	Drilling specific energy (DSE) features	Dolomite, shale, marl	R^2^ 0.8724 (*c*) R^2^ 0.8142 (*φ*)
ANFIS- GA^55^	Drilling specific energy (DSE) features	Dolomite, shale, marl	R^2^ 0.8154 (*c*) R^2^ 0.6914 (*φ*)
Support vector machine (SVM)^56^	P-wave velocity, Density, UCS, UTS	Limestone, quartzite, slate and quartz mica schist.	: (R^2^ = 0.977) and (R^2^ = 0.916)
Lasso regression (LR)^56^	P-wave velocity, Density, UCS, UTS	Limestone, quartzite, slate and quartz mica schist.	: R^2^ = 0.928 and : R^2^ = 0.606
Ridge regression (RR)^56^	P-wave velocity, Density, UCS, UTS	Limestone, quartzite, slate and quartz mica schist.	: R^2^ = 0.961 and : R^2^ = 0.822
Decision tree (DT)^56^	P-wave velocity, Density, UCS, UTS	Limestone, quartzite, slate and quartz mica schist.	: R^2^ = 0.934 and : R^2^ = 0.607

Using the triaxial test data from the Himalayan region, this study proposes a novel ML model combining the ISSA with XGBoost to predict internal friction angle and cohesion. The proposed model’s performance was evaluated by comparison with the XGBoost model (without ISSA tuning) and four other ML models. Furthermore, a feature importance analysis was conducted to support geotechnical engineers with limited ML expertise in interpreting the results. This study aims to provide a more efficient and reliable prediction of internal friction angle and cohesion, which is also the key to improve the quality of related building design.

## Dataset and preprocessing

### Data collection

The Himalayas are one of the youngest structurally active complex geological chains, and the physical and mechanical properties of the rock materials there show high uncertainty. A large number of rock engineering activities have been developed in the Himalayan region. Therefore, it is necessary to study the cohesion and internal friction angle of rock materials in this area to guide the design of rock structures.

**Fig. 2:**
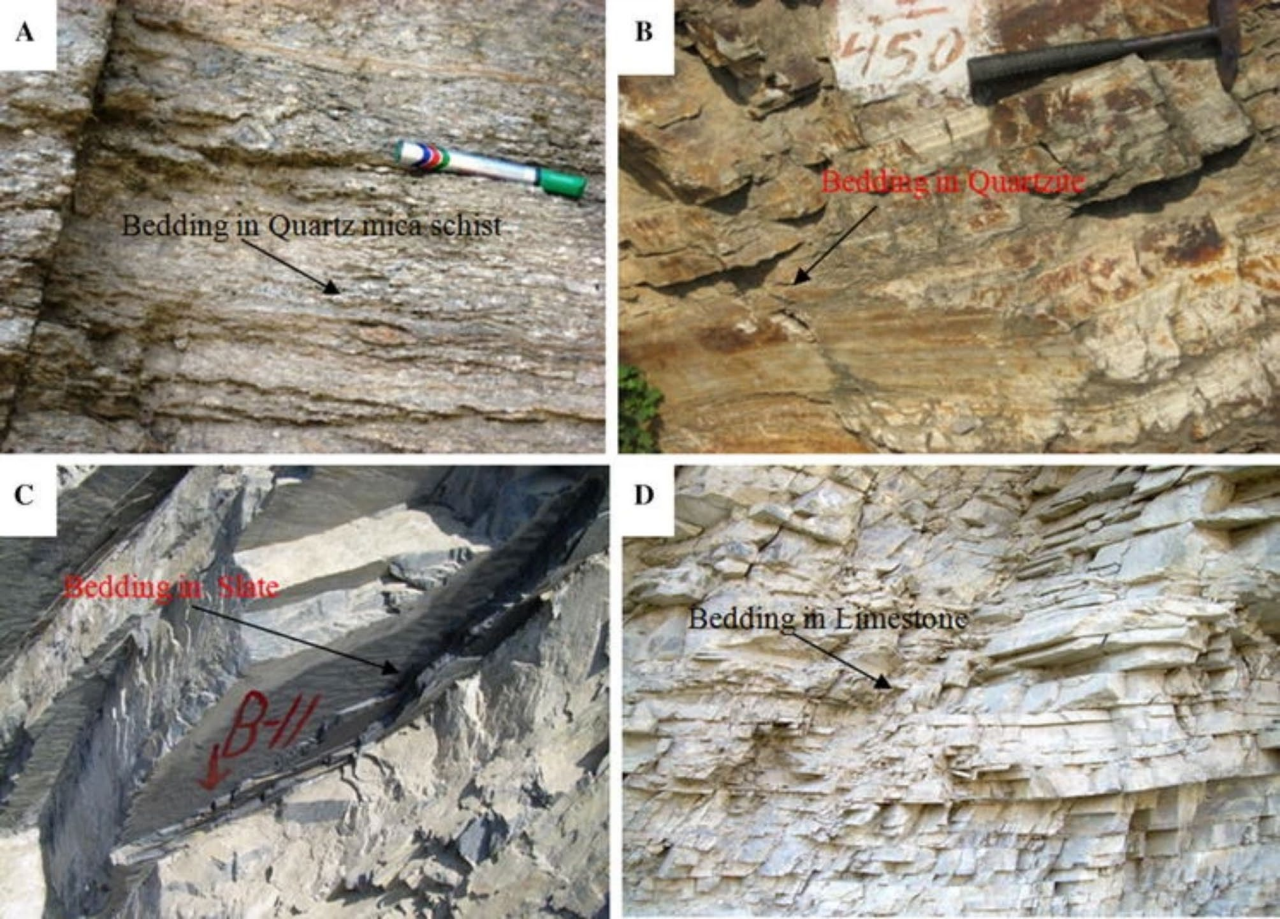
Four rock materials taken from the Luhri area, Himanchal Pradesh, India. (**a**) quartz mica schist (**b**) quartzite (**c**) slate (**d**) limestone^54^.

This paper collected experimental results from the literature^54^ as the dataset for developing ML models. As presented in Fig. 2, the rock materials tested in this dataset include limestone, quartzite, slate, and quartz mica schist collected from the Luhri area, Himanchal Pradesh, India. A total of 597 rock samples underwent tests, including uniaxial compression, tensile strength, triaxial testing, and longitudinal wave velocity measurements. The average of the three test results was analyzed as a single value, and finally 199 sets of valid data were obtained. Further details of these tests and rock samples can be found in the literature^54^. Previous study^57^ has demonstrated that incorporating four key mechanical properties—P-wave velocity, density, uniaxial compressive strength, and tensile strength (TS)—as input variables significantly enhances the predictive performance of ML models for shear strength parameters. Accordingly, these four properties were used as input variables in this study, with the shear strength parameters of rock materials (*φ* and *c*) designated as output variables.

### Data preprocessing

**Fig. 3:**
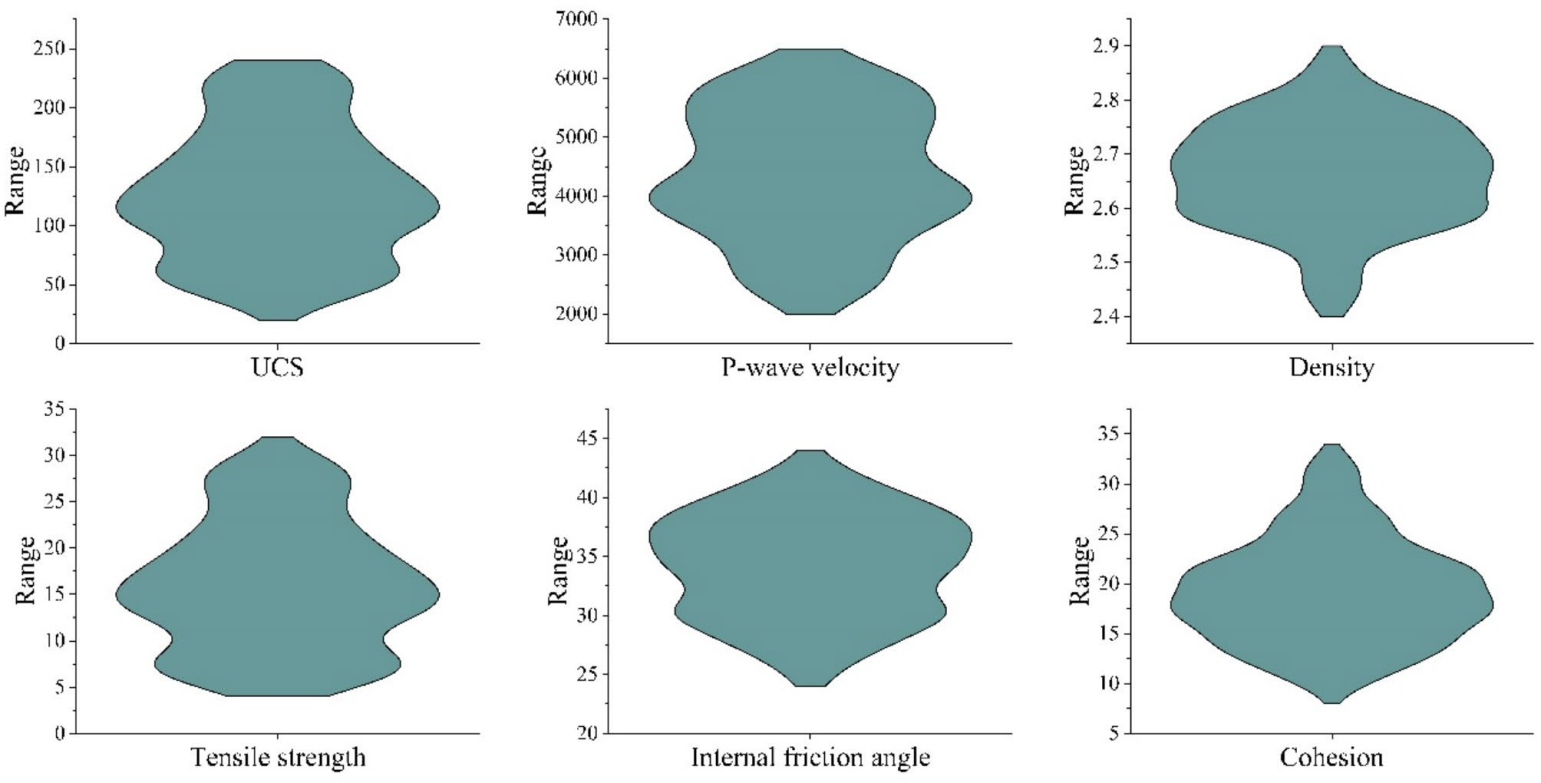
Violin plot of each parameter.

In general, the application of ML modeling requires data analysis and pre-processing. The statistical analysis of input and output variables in the dataset is shown in Fig. 3 and Table 2. The box diagram provides a comprehensive visual analysis of the input and output parameters (P-wave velocity, density, UCS, TS, *c*, *φ*) of the four rock types included in the dataset, showing the corresponding distribution characteristics. Table 2 complements this with detailed statistical metrics for each parameter. The analysis reveals that the six input and output variables across the four rock materials cover a wide value range, with no significant outliers detected.

**Table 2:** The detailed statistics of the input and output parameters.

	Skewness	Kurtosis	Coefficient of Variation	Minimum	Median	Maximum
P-wave velocity	−0.0683	−0.9759	0.2541	2209.34	4240.93	6328.14
Density	−0.2039	−0.2480	0.0368	2.41	2.67	2.89
UCS	0.2974	−0.8894	0.4336	40.97	120.9	237.76
TS	0.2911	−0.8727	0.4340	5.2	15.13	29.85
*c*	0.4770	−0.2314	0.2679	9.96	18.7	32.11
*φ*	−0.1208	−0.8783	0.129	24.57	34.55	43.35

The preprocessing of dataset mainly consists of normalized and segmented datasets. Normalization is the primary process for standardizing data in a dataset^58^. This step guarantees that all input variables are treated fairly when modeling and that no one parameter is overestimated or underestimated. Similar to unifying measurements of length—converting yards, inches, or feet to the standard unit of meters—normalization scales all input variables to a common range. This process enables the ML model to more effectively and efficiently capture complex nonlinear relationships. Normalization methods commonly used in ML include the minimum-maximum normalization method^59^, the Z-score normalization method^60^ and the robust scaling method^61^. In this paper, the minimum-maximum normalization method is used to re-scale the data to a range between 0 and 1. The mathematical formula for this method is provided in Eq. 1. The impact of alternative normalization techniques on model predictions is further investigated in the following section.

1 $$\bar {X}=\frac{{X - {X_{min}}}}{{{X_{max}} - {X_{\hbox{min} }}}}$$

where $X$ and $\bar {X}$ represent the original and normalized values respectively. *X*_*max*_ and *X*_*min*_ represent the maximum and minimum values of the original values, respectively.

Following is the data splitting of dataset. As a popular method for model validation, data splitting method randomly divides datasets into training set and test set^62^. The ML model is used on the training set to learn the training, by separating another part of the dataset independent of the training process (i.e., the test set) for validation^63^. Currently, there is no industry standard or specification for data segmentation ratio. Common data splitting ratios include 70:30 and 80:20. This represents 70 (80) percent of the dataset used for training and 30 (20) percent for testing. The predictive performance of the model can be effectively evaluated through data segmentation, which ensures that ML models are trained on representative samples while still being rigorously tested on new data that has never been seen before^64^. Therefore, in this paper, the data splitting ratio of 70:30 is used to randomly extract 139 data points and 60 data points from the dataset to create training and testing ML models respectively.

The data splitting method has potential shortcomings and biases in evaluating model prediction performance. In order to overcome these challenges, a 5-fold cross-validation method is implemented in this paper. As a popular statistical method, it provides a comprehensive and robust way to evaluate the predictive performance of ML models^65^. It not only reduces computation time, but also avoids any bias due to random data splitting (i.e. avoids underfitting and overfitting)^66^. In each fold, specify a different part for training and the rest for testing. This process is repeated in all folds until each part is utilized. Such a cross-validation process ensures that the ML model can fully learn on the training set. In the end, the best performing ‘optimal parameters’ are selected and passed on to the ML model to help it avoid overfitting.

Performance evaluation is an important part of the ML model^67^. Statistical evaluation indices are indispensable for quantifying the accuracy and reliability of model predictions. To evaluate the prediction performance, four commonly used statistical indices, defined in Eqs. 2–4 were used. Definitions and detailed statistical significance of these indicators can be found in the literatures^68,69^. In general, the prediction performance of the model is the best when these statistical evaluation indices reach the corresponding ideal value (R^2^ = 1, RMSE = 0, MAE = 0)^70^.

2 $${R^2}{\text{=1}} - \frac{{\sum_{{i=1}}^{N} {{{\left( {{S_o} - {S_p}} \right)}^2}} }}{{\sum_{{i=1}}^{N} {{{\left( {{S_o} - \overline {{{S_o}}} } \right)}^2}} }}$$

3 $$MAE=\frac{1}{N}\sum\limits_{{i=1}}^{N} {\left| {{S_o} - {S_p}} \right|}$$

4 $$RMSE=\sqrt {\frac{{\sum_{{i=1}}^{N} {{{\left( {{S_o} - {S_p}} \right)}^2}} }}{N}}$$

where *N* denotes the number of data. S_o_ and S_P_ are the actual and predicted results, respectively. $\overline {{{S_o}}}$ is the average of S_o_.

## Methodology

### Extreme gradient boosting

XGBoost is an advanced variant of gradient boosted decision tree (GBDT) proposed by Chen and Guestrin^71^. The algorithm reduces the error of the prediction of the previous step by continuously generating new regression trees, gradually reduces the error between the predicted value and the true value, and then improves the prediction effect of the model^72^. By providing parallel tree boosting, the model can solve nonlinear problems rapidly and accurately in an effective way, and has been widely used in several fields. In recent years, the concept of XGBoost has been introduced to nonlinear problems that require high precision. Details about the XGBoost can be easily found in the following papers^73–75^.

**Fig. 4:**
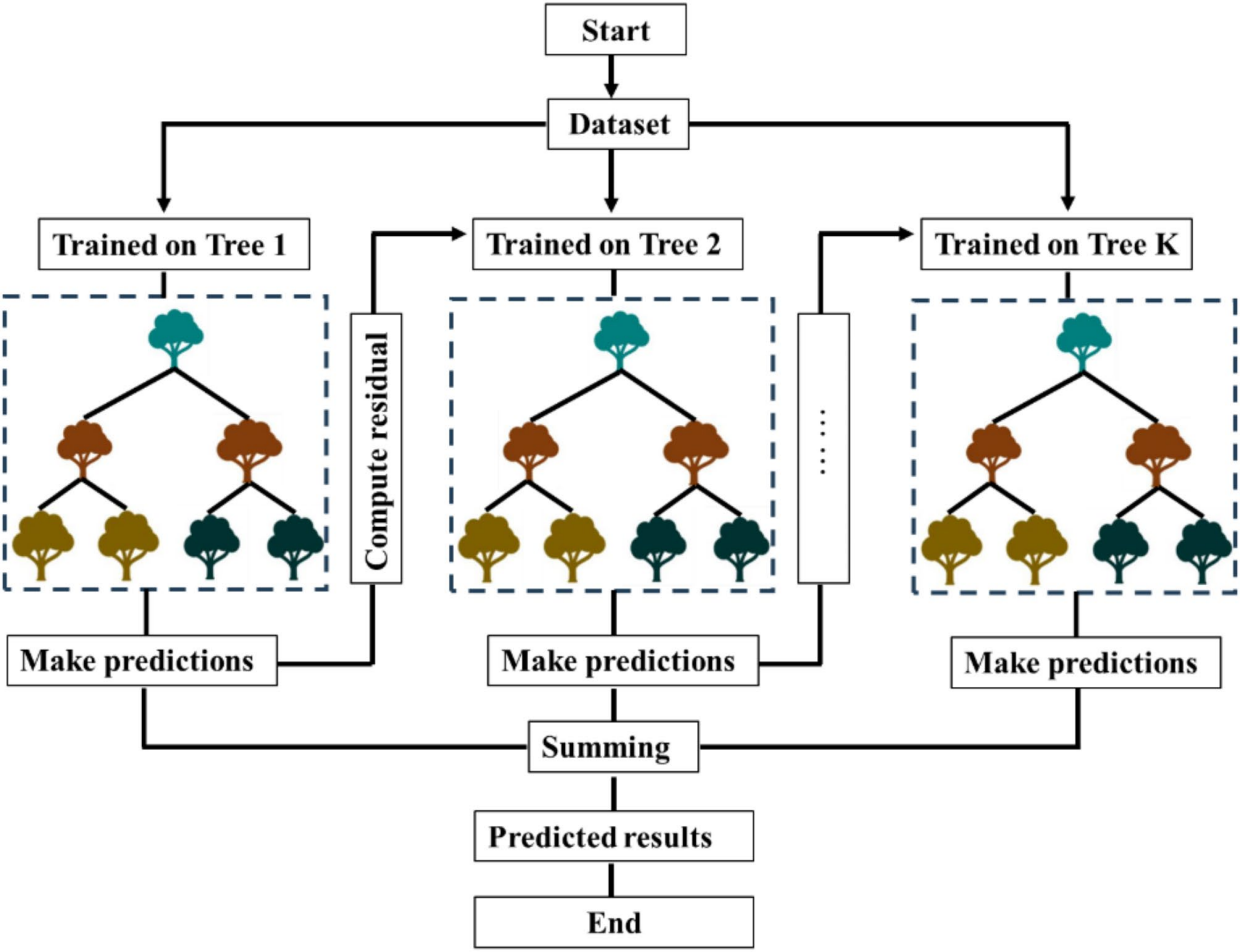
Graphical representation of XGBoost model.

The schematic of XGBoost is presented in Fig. 4. XGBoost uses a regression tree (CART) as the base learner. CART is a binary tree where each leaf node represents a numerical prediction, and each internal node signifies a conditional judgment based on eigenvalues. During the training process, XGBoost iteratively corrects model errors by adding new regression trees. The introduction of regularization and second-order gradient optimization enhances both the training efficiency and prediction accuracy of the model. Through continuous iterations, multiple low-precision trees are combined to form a high-precision predictive model.

The principle of the XGBoost algorithm is briefly described below and its prediction function is shown in Eq. 5.

5 $$\mathop {{y_i}}\limits^{ \wedge } =\sum\limits_{{k=1}}^{K} {{f_{\text{k}}}\left( {{x_i}} \right)} ,{f_{\text{k}}} \in \Gamma$$

where $\mathop {{y_i}}\limits^{ \wedge }$ is the predicted result value of the ith sample in the dataset, *K* is the total number of regression trees, *x*_*i*_ is the ith sample, and $\Gamma$ is the space of regression trees.

As shown in Eq. 6, the XGBoost adds a regular term $\Omega \left( {{f_{\text{k}}}} \right)$ (i.e., penalty function) to the objective function *Obj* to reduce overfitting and increase variety. Term $\sum_{{i=1}}^{n} {{\text{l}}\left( {{y_i},{{\mathop y\limits^{ \wedge } }_i}} \right)}$ in Eq. 6 characterizes the fit of the model, i.e. how well the predictions match the actual results, while term $\Omega \left( {{f_{\text{k}}}} \right)$ measures the complexity of the model.

6 $$Obj=\sum\limits_{{i=1}}^{n} {{\text{l}}\left( {{y_i},{{\mathop y\limits^{ \wedge } }_i}} \right)} +\sum\limits_{{k=1}}^{K} {\Omega \left( {{f_{\text{k}}}} \right)}$$

where *y*_*i*_ represents the true result value of the ith sample.

The penalty function can be rewritten as:

7 $$\Omega \left( {{f_k}} \right)=\frac{1}{2}\lambda \sum\limits_{{j=1}}^{T} {w_{j}^{2}} +\gamma T$$

where *γ* is the complexity cost of introducing additional leaf nodes, $w_{j}^{2}$ is the weight of the jth leaf node, λ is the regular term, and T is the number of leaf nodes.

The model was trained using the additive training method, as shown in Eq. 8. Additive training refers to the process of model training in which new tree models are gradually added to improve the overall model’s prediction ability by adjusting the prediction results of existing models. This process can also be understood as the gradient boosting method and is one of the core training ideas used by XGBoost.

8 $$Ob{j^{(t)}}=\sum\limits_{{i=1}}^{n} {{\text{l}}\left( {{y_i},\mathop {{y_i}}\limits^{{ \wedge (t - 1)}} +{f_{\text{k}}}\left( {{x_i}} \right)} \right)} +\Omega \left( {{f_{\text{k}}}} \right)$$

where $\mathop {{y_i}}\limits^{{ \wedge (t - 1)}}$ is the prediction for the (t-1)th sample at the ith iteration and *f*_*t*_ is used to reduce the loss function.

Equation 8 is optimized with a 2nd-order Taylor expansion to obtain the final objective function as shown in Eq. 9. The parameters are continuously updated through Eq. 9 until the conditions are satisfied.

9 $$Ob{j^{(t)}}=\sum\limits_{{k=1}}^{K} {\left[ {{g_i}{f_k}\left( {{x_i}} \right)+\frac{1}{2}{h_i}{{\left( {{f_k}\left( {{x_i}} \right)} \right)}^2}} \right]} +\Omega \left( {{f_{\text{k}}}} \right)$$

where ${g_i}$ and ${h_i}$ denote the first and second derivatives obtained from the loss function, respectively.

### Improved sparrow search algorithm

The sparrow search algorithm (SSA), a meta-heuristic machine learning algorithm, represents an innovative development in population intelligence optimization techniques^76^. SSA achieves parameter optimization by emulating natural sparrow behaviors, such as foraging and anti-predation strategies. Compared to other population intelligence algorithms, SSA is distinguished by its robust optimization capabilities and exceptional stability, making it widely applicable across diverse fields, including engineering, mathematics, and computer science^77–79^.

**Fig. 5:**
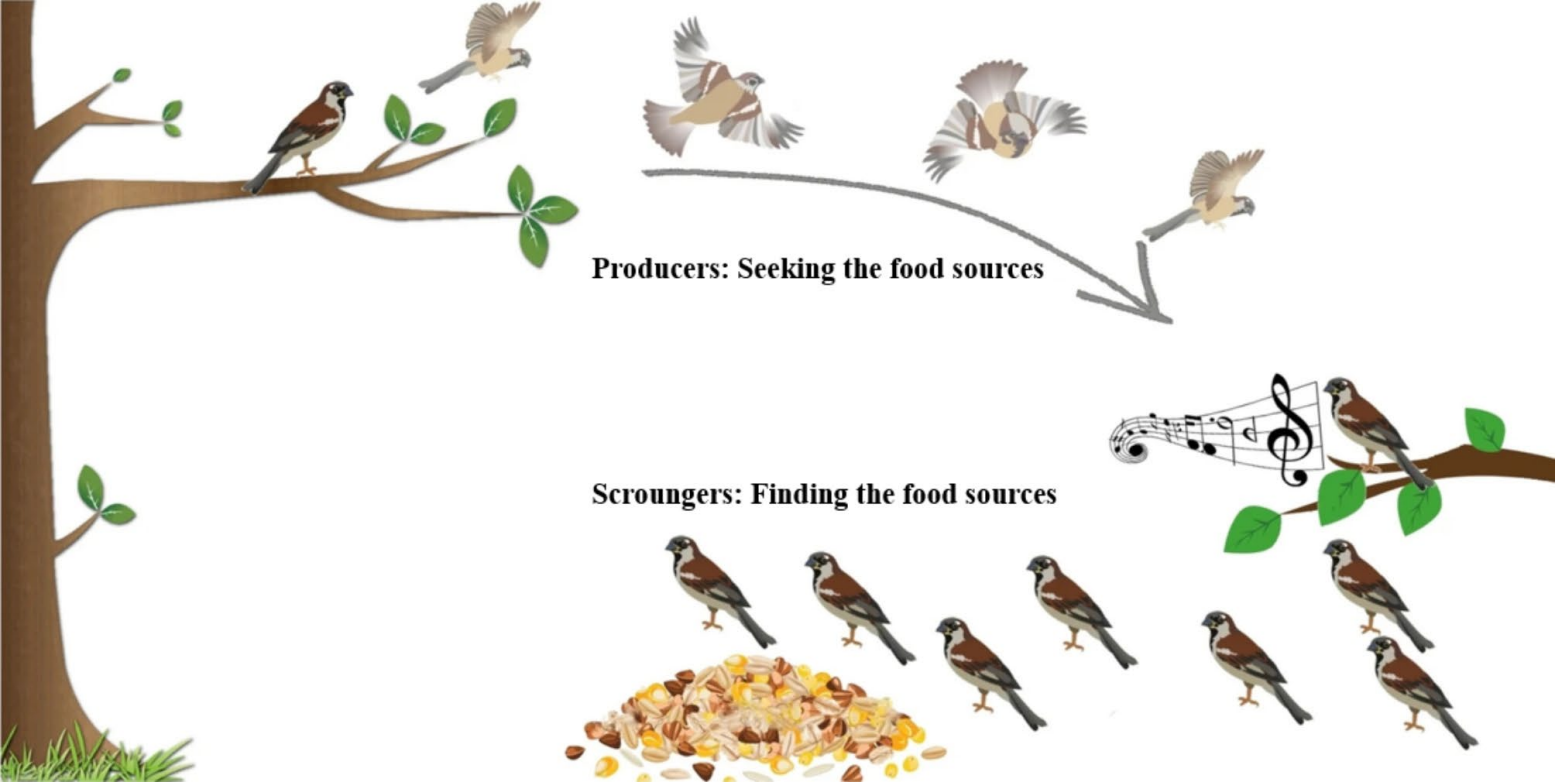
Graphical representation of sparrow search algorithm (SSA), modified from^77^.

Depending on the classification, sparrow populations contain both producers and scroungers. As shown in Fig. 5, producers are responsible for locating food and guiding the population to food sources, while scroungers depend on the producers to access these resources. The process is outlined as follows:

As shown in Eq. 10, a sparrow population can be mathematically represented as a two-dimensional matrix of size *N* x *D*. Each element in Eq. 10 represents the decision variable at the *j*th position of the *i*th sparrow, and each row vector represents the set of sparrows at the *i*th position.

10 $$P{\text{opulation}}=\left[ {\begin{array}{*{20}{c}} {{x_{1,1}}}&{{x_{1,2}}}&{\cdots}&{{x_{1,D}}} \\ {{x_{2,1}}}&{{x_{2,2}}}&{\cdots}&{{x_{2,D}}} \\ {\cdots}&{\cdots}&{\cdots}&{\cdots} \\ {{x_{M,1}}}&{{x_{M,2}}}&{\cdots}&{{x_{M,D}}} \end{array}} \right]$$

where *M* is the population size and *D* is the dimension of the search space.

Each *x*_*i, j*_ is given a random value within the specified upper and lower bound. The fitness function is employed to compute the fitness value for each sparrow location in the population and determine the location of the best sparrow (e.g., *x*^*Gbest*^) in the population. The sparrow with the better fitness value is prioritized for food and acts as a producer to lead the entire population to run to the food source.

As shown in Eq. 11, the location of producers is updated to^80^:

11 $$x_{{{\text{i,j}}}}^{{g+1}}=\left\{ {\begin{array}{*{20}{c}} {x_{{{\text{i,j}}}}^{g}+Q \times L}&{r \geqslant ST} \\ {x_{{{\text{i,j}}}}^{g} \times {e^{\frac{{ - i}}{{\alpha \times {G_{\hbox{max} }}}}}}}&{r< ST} \end{array}} \right.$$

where *g* represents the current number of iterations, *G*_*max*_ is the maximum number of iterations considered in the search process; *Q* is a random number obeying a normal distribution, α is a uniform random number in (0, 1); the alarm threshold *r* belongs to [0, 1], the safety threshold *ST* belongs to [0.5, 1], and *L* is a matrix that is 1 × d and has elements assigned to value 1.

As shown in Eq. 12, the location of scroungers is updated to:

12 $$x_{{{\text{i,j}}}}^{{g+1}}=\left\{ {\begin{array}{*{20}{c}} {x_{j}^{{Pbest}} - \left| {x_{{{\text{i,j}}}}^{g} - x_{j}^{{Pbest}}} \right| \times {A^+} \times L}&{i \leqslant \frac{M}{{\text{2}}}} \\ {Q{e^{\frac{{x_{j}^{{{\text{worst}}}} - x_{{{\text{i,j}}}}^{g}}}{{{i^2}}}}}}&{i> \frac{M}{{\text{2}}}} \end{array}} \right.$$

where $x_{j}^{{Pbest}}$and $x_{j}^{{W{\text{orst}}}}$ are the best and worst positions of the discoverer, respectively, and *A*^*+*^ is a 1 × d matrix whose elements are randomly assigned values − 1 and 1.

When the sparrow is foraging, a randomly selected portion of the population (usually taken as 10–20%) of sparrows will be responsible for vigilance. In a dangerous situation, either the producer or the scrounger will abandon the current food and fly to a new safe location. The positions of these scouters are updated as shown in Eq. 13.

13 $$x_{{{\text{i,j}}}}^{{g+1}}=\left\{ {\begin{array}{*{20}{c}} {x_{{{\text{i,j}}}}^{g}+K\left( {\frac{{\left| {x_{{{\text{i,j}}}}^{g} - x_{j}^{{{\text{worst}}}}} \right|}}{{\left( {f\left( {x_{{\text{i}}}^{g}} \right) - f\left( {x_{{}}^{{{\text{worst}}}}} \right)} \right)+\varepsilon }}} \right)}&{f\left( {x_{{\text{i}}}^{g}} \right){\text{=}}f\left( {{x^{Gbest}}} \right)} \\ {x_{j}^{{Gbest}}+\beta \times \left| {x_{{{\text{i,j}}}}^{g} - x_{j}^{{Gbest}}} \right|}&{f\left( {x_{{\text{i}}}^{g}} \right)> f\left( {{x^{Gbest}}} \right)} \end{array}} \right.$$

where $f\left( {x_{{\text{i}}}^{g}} \right)$ is the fitness value of the ith sparrow at the gth iteration, and $f\left( {x_{{}}^{{{\text{worst}}}}} \right)$ is the fitness value of the worst sparrow in the population; *β* is a step control parameter; *K* is a random number ranging from − 1 to 1, and $\varepsilon$ is a small random value to avoid the denominator to be 0. ${x^{Gbest}}$ is the best-positioned sparrow in the population.

Although the SSA algorithm has significant advantages in terms of search accuracy, stability and convergence speed, it still has some serious flaws. For example, the convergence strategy of SSA is to jump directly to the neighborhood of the current optimal solution, which will be underpowered at the late stage of the search for optimality and underpowered for local search. To solve this problem, the Lévy flight strategy is introduced into the location of scroungers to improve the global search capability. As shown in Eq. 14, the introduction of the Lévy flight strategy modifies Eq. 12. This optimized algorithm is known as the Improved sparrow search algorithm (ISSA).

14 $$x_{{{\text{i,j}}}}^{{g+1}}=\left\{ {\begin{array}{*{20}{c}} {x_{j}^{{Pbest}}+H\left| {x_{{{\text{i,j}}}}^{g} - x_{j}^{{Pbest}}} \right| \times {A^+} \times L}&{i \leqslant \frac{M}{{\text{2}}}} \\ {Q{e^{\frac{{x_{j}^{{{\text{worst}}}} - x_{{{\text{i,j}}}}^{g}}}{{{i^2}}}}}}&{i> \frac{M}{{\text{2}}}} \end{array}} \right.$$

where *H* is a random number determined by Eqs. 15–17.

15 $$H=\mu /{\left| {\text{v}} \right|^{1/\delta }},\delta =\frac{3}{2}$$

16 $$\mu \sim N\left( {0,\sigma _{\mu }^{2}} \right),v\sim \left( {0,\sigma _{v}^{2}} \right)$$

17 $$\sigma _{\mu }^{{}}={\left\{ {\frac{{\Gamma \left( {1+\delta } \right)\sin \left( {\frac{{\pi \delta }}{2}} \right)}}{{\delta \Gamma \left[ {\frac{{1+\delta }}{2}} \right]{2^{\frac{{\delta - 1}}{2}}}}}} \right\}^{1/\delta }},\sigma _{v}^{{}}=1$$

### Model development

Generally, ML models that do not incorporate optimization algorithms often have convergence problems. Moreover, the artificial determination of model hyperparameters is subjective and unfavorable for application. XGBoost model improves the computing speed and accuracy to the extreme on the basis of efficient implementation of the gradient boosting decision tree algorithm, but the step-by-step growth strategy leads to unnecessary consumption of computer operating resources. The construction process of the ML model essentially lies in the determination of hyperparameters^81^. In this section, the ISSA algorithm is used to optimize the hyperparameters of the XGBoost model. Accordingly, a new hybrid ML model (i.e., ISSA-XGBoost model) is established. To the best of the authors’ knowledge, the application of the ISSA algorithm in improving hybrid ML models for predicting cohesion and internal friction angle has not been reported yet.

**Fig. 6:**
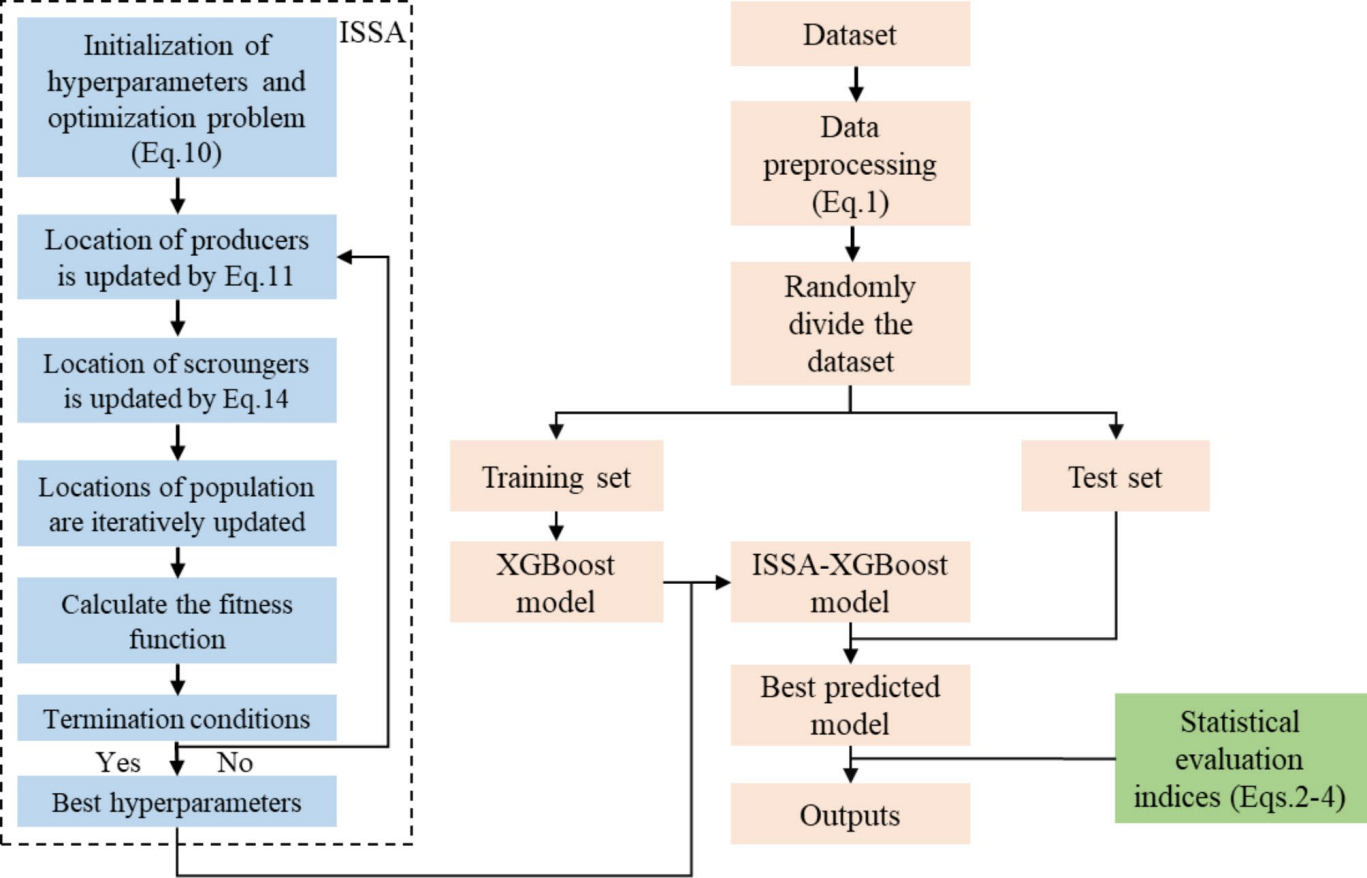
Framework example of the proposed ISSA-XGBoost model.

The modeling steps of the hybrid ISSA-XGBoost model are outlined in Fig. 6. After collecting the raw dataset, the raw data are processed using a suitable normalization method (e.g., Eq. 1). Randomly divide the dataset into the test set and training set. Then, the ISSA algorithm is initialized and the search space as well as the model hyperparameters are set. Set the range of parameters to be optimized in the XGBoost model to generate the initial population of sparrows. Next, based on the resulting sparrow population, iterate with statistical evaluation indices such as R^2^ or RMSE as the fitness function to calculate the positional fitness of each sparrow. The obtained fitness values are sorted and the current global best position is localized. The sparrow position is continuously updated according to Eqs. 10–17. Finally, when the number of iterations satisfies the termination condition, terminate the iteration and output the parameters corresponding to the best sparrow position.

## Results and analysis

### Model performance

**Fig. 7:**
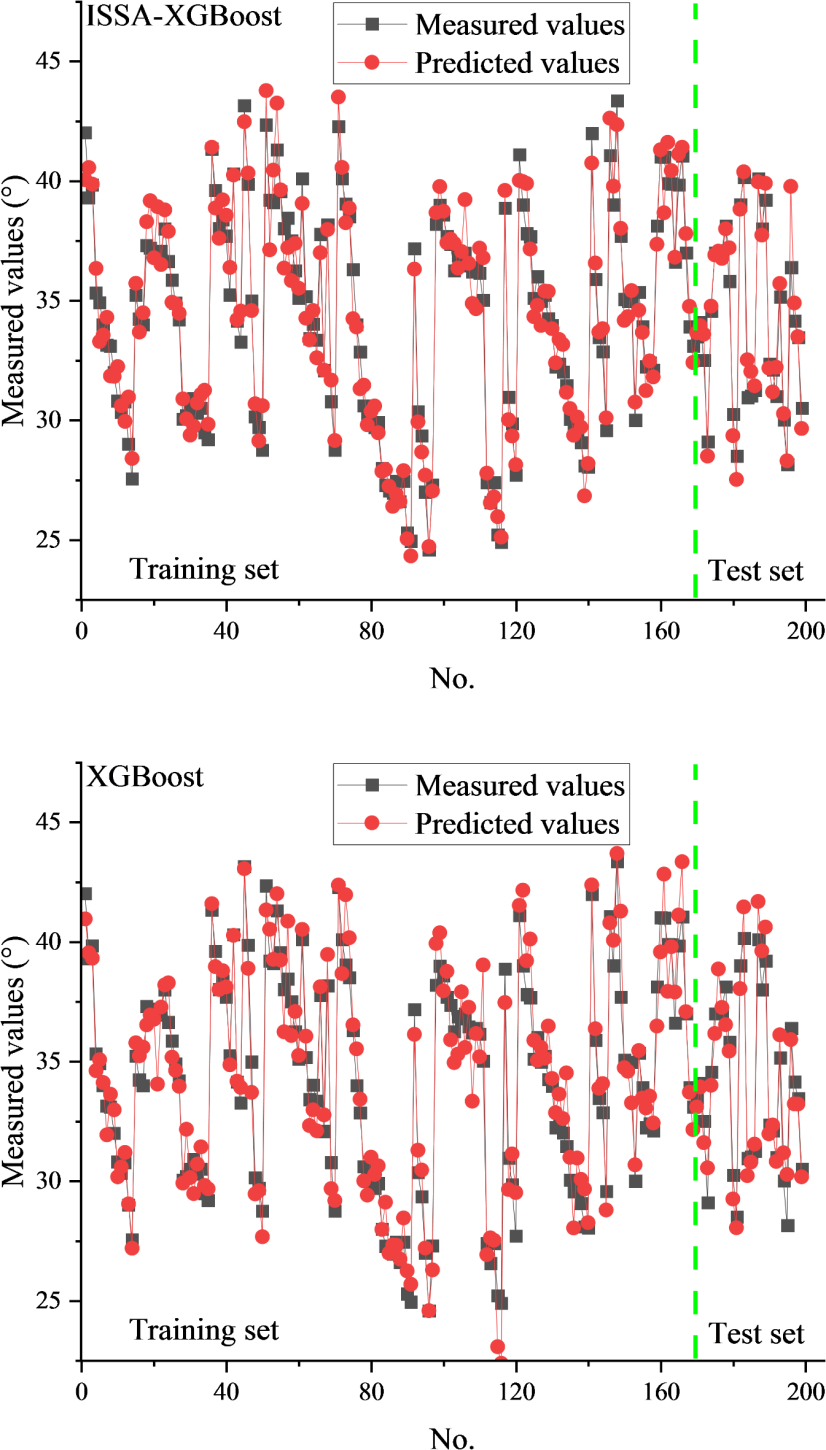
Comparisons between the predicted and measured internal friction angles.

**Fig. 8:**
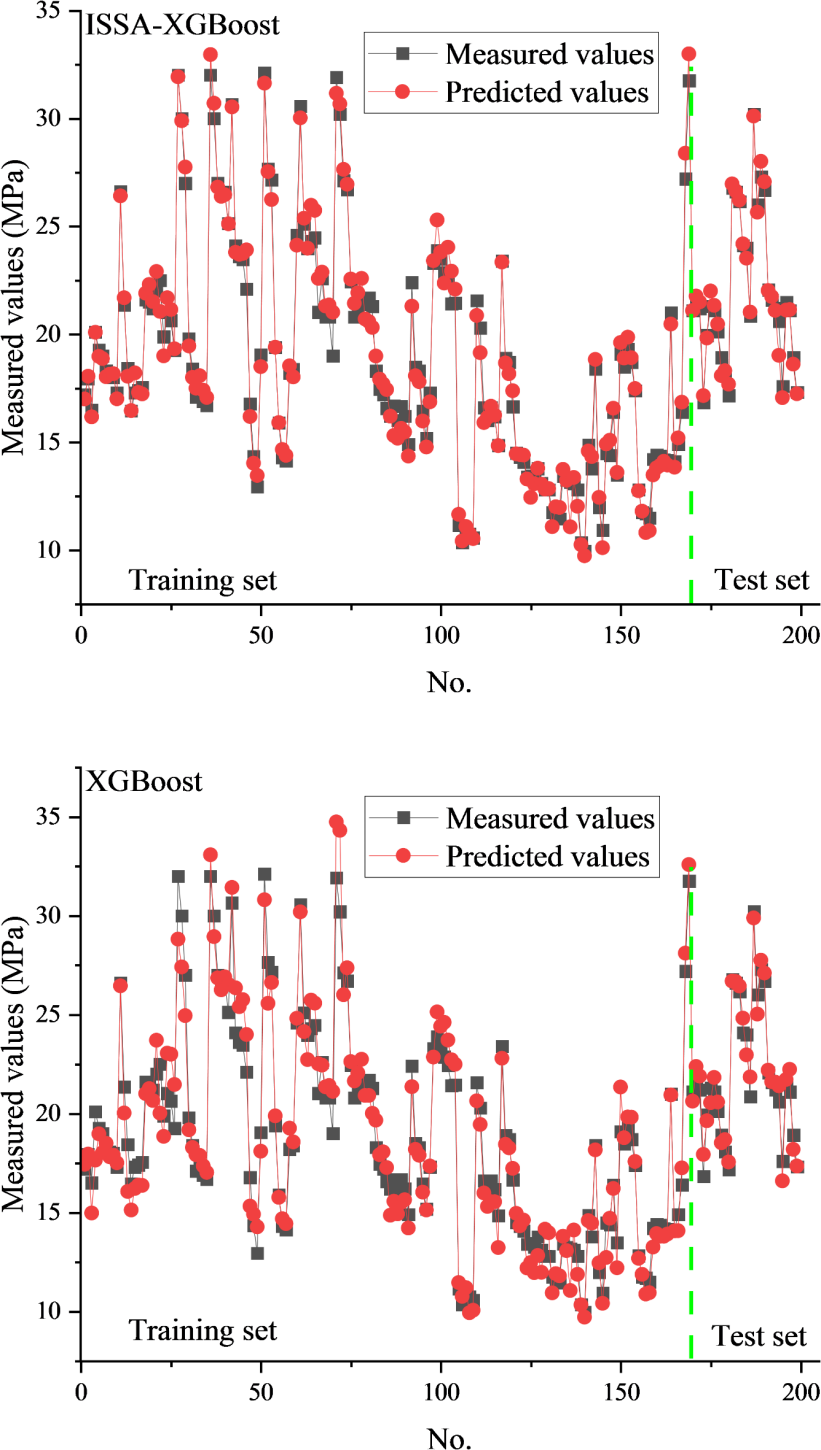
Comparisons between the predicted and measured cohesions.

For comparison, the predictions of the XGBoost model are also presented. In this case, the XGBoost model was only validated with 5-fold cross-validation method and did not use any optimization algorithm for hyperparameter optimization. The prediction performance of the proposed hybrid ML model and the XGBoost model for both the training and test sets is illustrated in Figs. 7 and 8.

Each point in Figs. 7 and 8 represents a predicted sample, with the X-axis denoting the data index and the Y-axis representing either the cohesion or internal friction angle. From these figures, it can be seen that the prediction results of the ISSA-XGBoost model are closer to the experimental results both on the training set and the test set. Compared with the XGBoost model, the ISSA-XGBoost model is closer to the experimental results. This indicates that the ISSA-XGBoost model is very accurate in modeling the internal friction angle and cohesion. Table 3 shows the statistical evaluation indices of the two ML models on the training set and test set, respectively. It can be seen that for both cohesion and internal friction angle, the R^2^ values on the training set are very close to the R^2^ values on the test set. This result indicates that the ISSA-XGBoost model is well-trained. It is reasonable that the R^2^ values on the test set are slightly lower. Regarding cohesion and internal friction angle, the ISSA-XGBoost model significantly outperforms the XGBoost model (on both the training and test sets). The excellent prediction accuracy, as demonstrated in Table 3, underscores the potential of the proposed hybrid ML model as a reliable tool for predicting cohesion and the internal friction angle.

**Table 3:** Prediction performances for each model.

Parameters	Model	Dataset	*R* ^2^	RMSE	MAE
Internal friction angle	ISSA-XGBoost	Training set	0.957	0.943	0.770
		Test set	0.932	0.920	0.625
	XGBoost	Training set	0.931	1.192	0.922
		Test set	0.919	1.002	0.831
Cohesion	ISSA-XGBoost	Training set	0.985	0.473	0.639
		Test set	0.982	0.322	0.450
	XGBoost	Training set	0.953	0.895	1.135
		Test set	0.964	0.549	0.642

### Comparison with previous studies

In this section, the proposed ML model is evaluated against four other ML models: the lasso regression (LR), the ridge regression (RR), the support vector machine (SVM), and the decision tree (DT). After training on a dataset split into 70% training and 30% test sets, the models were evaluated on the test set. The three statistical evaluation indices of each model are shown in Table 4. For cohesion, the differences in prediction performance among the models are relatively small. The best performer is the proposed model of this paper, whose three statistical evaluation indices are RMSE = 0.322, MAE = 0.450, R^2^ = 0.982. For the internal friction angle, the proposed model significantly outperforms the other four models. Compared with the worst-performing LR model (R^2^ = 0.606, RMSE = 2.7255, MAE = 2.3064), the three statistical indicators of the proposed model are as high as R^2^ = 0.932, RMSE = 0.920, MAE = 0.625. In conclusion, the proposed model demonstrates superior performance, not only compared to the four alternative ML models but also relative to the XGBoost model.

**Table 4:** Prediction performances for each model.

Model		RMSE	MAE	*R* ^2^
LR	Internal friction angle	2.7255	2.3064	0.606
	Cohesion	1.4896	1.1454	0.928
RR	Internal friction angle	2.7256	2.3003	0.607
	Cohesion	1.2412	1.0335	0.934
SVM	Internal friction angle	1.2906	0.9094	0.916
	Cohesion	0.8253	0.5577	0.977
DT	Internal friction angle	2.2963	1.7655	0.822
	Cohesion	1.0560	0.8389	0.961
ISSA-XGBoost model	Internal friction angle	0.920	0.625	0.932
	Cohesion	0.322	0.450	0.982

## Discussion

### Data preprocessing methods

Data preprocessing methods play a critical role in maintaining data consistency and integrity, enabling ML models to achieve optimal predictive performance. The effectiveness of ML models depends heavily on the chosen algorithm and dataset. Once these factors are established, data preprocessing becomes a decisive element in influencing model performance. Proper data preprocessing not only eliminates magnitude discrepancies in raw data—avoiding issues such as the “big numbers eat decimals” phenomenon—but also significantly enhances the computational efficiency of ML models^82^. In this section, we will discuss the effect of three common data preprocessing methods on the ML model performance.

The minimum-maximum normalization method, zero-mean normalization method, and arctangent normalization method were selected to evaluate the effects of different data preprocessing techniques on the predictive performance of the ISSA-XGBoost model. The formula for the minimum-maximum normalization method is presented in Eq. 1, while the zero-mean normalization and arctangent normalization methods are detailed in Eqs. 18 and 19. Equation 18 transforms the raw dataset into a standard normal distribution with unit standard deviation and zero mean. And Eq. 19 transforms the raw dataset to range [−1,1].

18 $$\bar {X}=\frac{{X - \mu }}{\sigma }$$

19 $$\bar {X}={\text{2}}\frac{{\arctan X}}{\pi }$$

where *µ* and *σ* are the mean and standard deviation of the dataset, respectively.

**Fig. 9:**
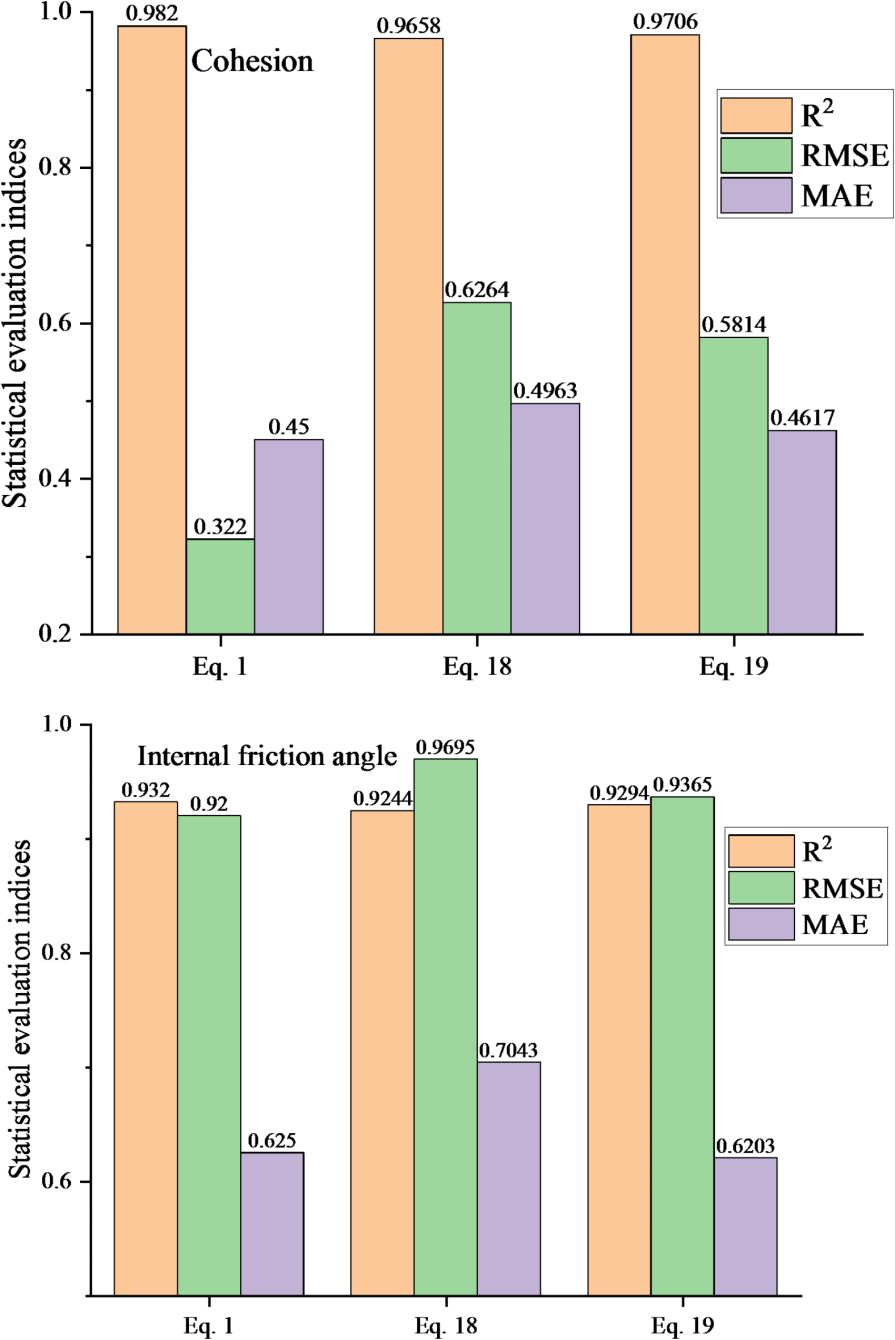
Predictive indicator results for different data preprocessing method.

The original dataset was processed using Eqs. 18 and 19 before undergoing the same modeling process. The statistical evaluation indices for each model are presented in Fig. 9. The results indicate that all three data preprocessing methods achieve predictions closely aligned with the actual values, demonstrating the ISSA-XGBoost model’s capability to accurately simulate the shear strength parameters of rocks. Among the preprocessing methods, Eq. 1 delivers significantly higher accuracy compared to Eqs. 18 and 19. Notably, even the method with the lowest prediction accuracy (Eq. 18 in Fig. 9) outperforms the four ML models listed in Table 4 in predicting both internal friction angle and cohesion.

### Feature importance score

**Fig. 10:**
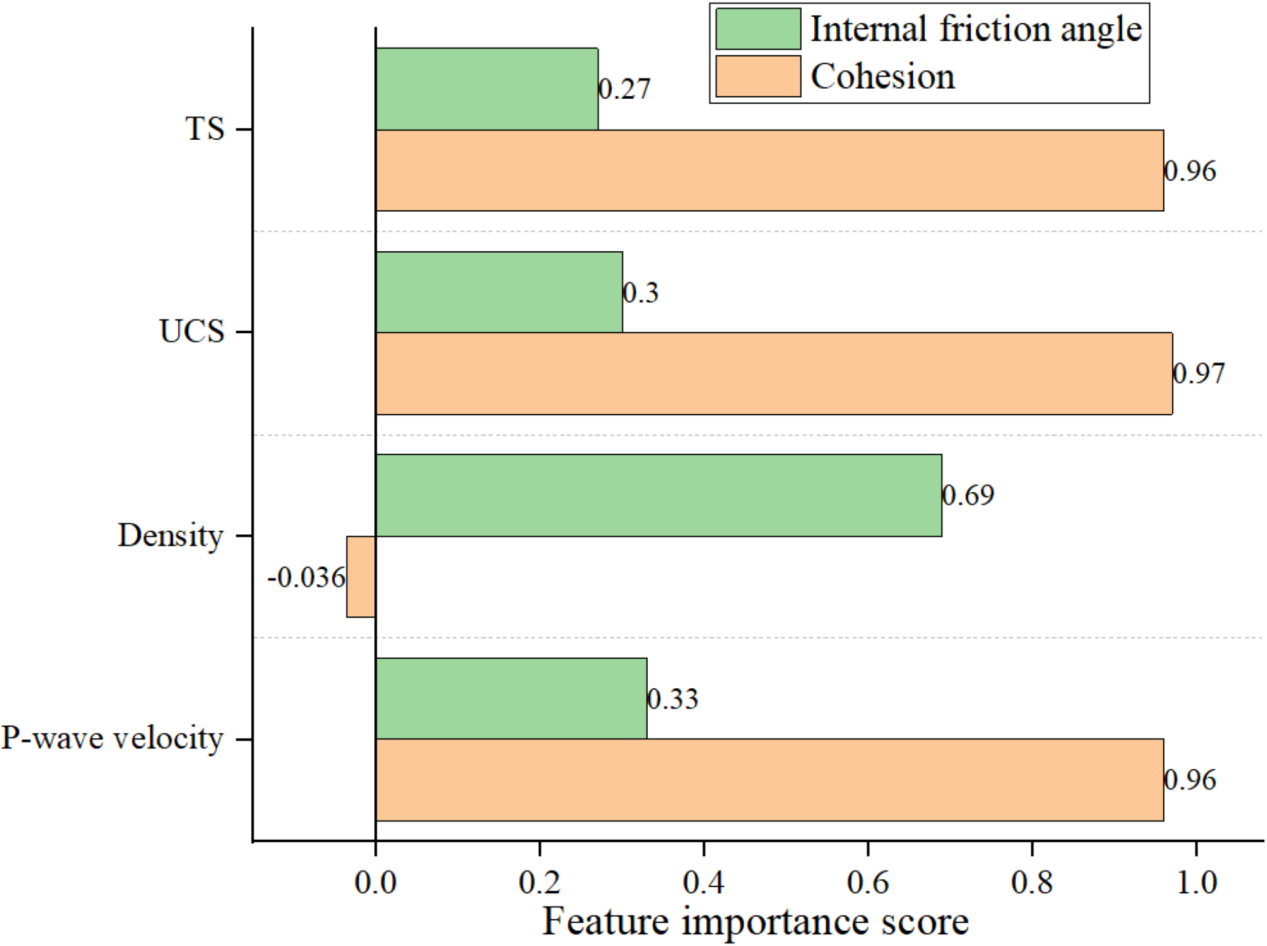
Feature importance score of inputs.

Feature importance analysis serves as a critical reference for assessing the contribution of input parameters to the model’s predictions^83,84^. Figure 10 shows the feature importance score of each input variable in the developed model to cohesion and internal friction angle. A higher feature importance score indicates a relatively greater influence of the corresponding input variable on the output variable^85^. The results reveal that the input variables exert different impacts on the two properties. For the cohesion, the effects of the four input variables are 0.96 (P-wave velocity), −0.036 (density), 0.97 (UCS), 0.96 (TS). For the internal friction angle, the scores 0.33 (P-wave velocity), 0.69 (density), 0.3 (UCS), 0.27 (TS). Among the input parameters, UCS demonstrates the greatest relative importance for cohesion, whereas density has the highest relative importance for the internal friction angle.

### Limitations

Internal friction angle and cohesion hold significant potential for economic benefits such as cost optimization and reduced time investment when determined using ML techniques. This paper introduces a novel ML model that integrates the strengths of XGBoost and ISSA, providing a reliable hybrid approach for predicting internal friction angle and cohesion. Although this paper has yielded valuable insights, its limitations should not be overlooked. For example, errors inherent to ML models and the variability introduced by experimental results are unavoidable^86,87^. In practical engineering, moisture content has a great influence on internal friction angle and cohesion^88,89^. However, due to the absence of moisture content data in the compiled dataset, it was not included as an input variable in the proposed ML model. Despite this omission, the model still achieves satisfactory predictive performance. A possible explanation is that, within the selected dataset described in Sect. 2, the moisture content of the samples is consistent. Consequently, the ML model delivers reliable predictions even without moisture content as an input.

Furthermore, as more data becomes available, the generalization ability and prediction accuracy of the constructed ML model can be further enhanced^90^. In future research, expanding the dataset to include data from various types of rock materials will be a valuable step. Additionally, the input variables, such as uniaxial compressive strength and tensile strength, currently have a limited value range. Expanding these ranges could improve the model’s generalization capability. Lastly, to bridge the gap between computational predictions and practical applications, we aim to develop a user-friendly graphical user interface.

## Conclusions

1. Utilizing ML technology, cohesion and internal friction angle of rock materials can be accurately estimated using extensive historical data. The proposed model, built on four input variables, demonstrates strong generalization ability and high prediction accuracy. Compared with the actual observed values, the new model gives reliable prediction results with R^2^, RMSE and MAE values of 0.932(*φ*), 0.982(*c*), 0.920(*φ*), 0.322(*c*), 0.625(*φ*), 0.450(*c*), respectively.
2. The proposed ISSA-XGBoost model was compared with five other ML models. For parameter internal friction angle, the performance ranking is ordered as ISSA-XGBoost > XGBoost > SVM > DT > RR > LR. For parameter cohesion, the ranking is slightly different: ISSA-XGBoost > SVM > XGBoost > DT > RR > LR.
3. The feature importance analysis shows that the four input parameters affect cohesion in the following order from strongest to weakest: UCS > P-wave velocity = TS > density. The four input parameters affect the internal friction angle in the following order from strongest to weakest: density > P-wave velocity > UCS > TS.

## Data Availability

The data used to support the findings of this study are available from the corresponding author upon request.
